# O.2.2-7 Pilot study of a health promotion intervention in sports clubs: evaluation of the implementation of the PROSCeSS intervention

**DOI:** 10.1093/eurpub/ckad133.122

**Published:** 2023-09-11

**Authors:** Benjamin Tezier, Aurélie Van Hoye, Anne Vuillemin, Fabienne Lemonnier, Florence Rostan, Francis Guillemin

**Affiliations:** Université de Lorraine, APEMAC, France; Université de Lorraine, APEMAC, France; University of Limerick, PfAH Research Cluster, Ireland; Université Côte d’Azur, LAMHESS, France; Department of Health Promotion, Santé Publique France, France; Benjamin.tezier@univ-lorraine.fr; Department of Health Promotion, Santé Publique France, France; Benjamin.tezier@univ-lorraine.fr; Université de Lorraine, APEMAC, France

## Abstract

**Purpose:**

Given the health promotion (HP) potential of the sport club (SC) and the paucity of HP interventions rigorously evaluated in this setting, a HP intervention was co-constructed from the Health Promoting SC theoretical framework to enable the application of a setting based approach. The present pilot study investigates the implementation of this intervention and how it is influenced by the characteristics of SCs.

**Methods:**

A qualitative study involving recordings, observations and interviews was conducted with 14 SCs. Implementation of HP strategies and intervention steps was informed by the implementation traces collected during meetings, emails and calls. The influence of SCs characteristics on the implementation of the intervention and strategies was elucidated through semi-structured interviews with the project leaders of each SC. Data were analyzed based on the HPSC model.

**Results:**

4 SCs have implemented between 9 and 13 multi-level and multi-determinant strategies that allow them to develop adapted sports sessions and educational activities nutrition, warm-up and first aid. Their ability to develop HP activities and implement intervention steps is influenced by the human resources available, the support they receive from the sports federations and the ability of a referent to get involved. Identifying SC experiences and resources to promote health and communicating HP activities were the most used strategies. The results and feedback from the project leaders enabled the intervention steps to be reworked and an evaluation design to be developed for future work.

**Conclusion:**

This study highlights the importance of taking into consideration the needs and specificities of the contexts in which HP interventions are deployed, as well as the importance of piloting interventions. The results highlight in particular the relevance of using the setting based approach to develop a global and sustainable approach to health in SCs.

**Funding:**

The work was supported through a partnership between Santé Publique France, Université Côte d’Azur and the Université de Lorraine and through a doctoral grant from the Grand-Est Region and the Pole Biologie-Medecine-Santé of Université de Lorraine.

